# Reduction of gastric cancer proliferation and invasion by miR-15a mediated suppression of Bmi-1 translation

**DOI:** 10.18632/oncotarget.7392

**Published:** 2016-02-15

**Authors:** Changping Wu, Xiao Zheng, Xiaodong Li, Andrew Fesler, Wenwei Hu, Lujun Chen, Bin Xu, Qi Wang, Anthony Tong, Stephanie Burke, Jingfang Ju, Jingting Jiang

**Affiliations:** ^1^ Department of Oncology, The Third Affiliated Hospital of Soochow University, Changzhou, China; ^2^ Department of Biological Treatment, The Third Affiliated Hospital of Soochow University, Changzhou, China; ^3^ Jiangsu Engineering Research Center for Tumor Immunotherapy, Changzhou, China; ^4^ Translational Research Laboratory, Department of Pathology, Stony Brook University, Stony Brook, NY, USA; ^5^ BioGenex Inc., Fremont, CA, USA

**Keywords:** miR-15a, gastric cancer, Bmi-1, survival

## Abstract

B-cell specific moloney leukemia virus insertion site 1 (Bmi-1) gene plays important roles in gastric cancer, but the epigenetic regulatory mechanism by microRNA (miRNA) and the functional significance of Bmi-1 inhibition in gastric cancer remains elusive. In this study, we systematically investigated the functional roles of miRNA mediated Bmi-1 suppression in gastric cancer. Our results show that the expression of miR-15a is significantly reduced in gastric cancer and the protein expression levels of Bmi-1 are inversely correlated with miR-15a (*P* = 0.034) in gastric cancer patient samples. Functional studies revealed that ectopic expression of miR-15a decreased Bmi-1 in gastric cancer cell lines with reduced proliferation and tumor invasion. High levels of Bmi-1 in gastric cancer patients are significantly associated with better overall survival (*P* = 0.024) based on the Kaplan-Meier survival analysis.

## INTRODUCTION

Gastric cancer is the fifth most common malignancy globally and the third leading cause of cancer-related deaths [1, 2]. Despite the treatment progress and research efforts in gastric cancer, the outcome of patients with stage IV disease remains disappointing, with a 5-year overall survival rate of 4% [3].

Gastric cancer is a genetic disease and multiple factors are involved in the multi-step processes of gastric cancer development [4, 5]. The majority of gastric cancers are associated with bacterium *Helicobacter pylori* (*H. Pylori*) and Epstein-Barr virus (EBV) [6]. The molecular and clinical characteristics are quite complex with both histological and aetiological heterogeneity. Based on The Cancer Genome Atlas (TCGA) analysis, it was proposed that gastric cancer can be divided into 4 unique subtypes [7]. The importance of epigenetic alterations in gastric cancer development and progression has only recently been appreciated [8]. One crucial aspect of epigenetic regulations is the involvement of non-coding miRNAs in gastric cancer development and progression [9].

miRNAs are non–protein-coding small RNAs with length between 19 and 25 nucleotides cleaved from 70- to 100- nucleotide hairpin pre-miRNA precursors [10]. miRNAs post-transcriptionally regulate protein expression of many target genes by interacting with the 3′-UTR region of the mRNA transcripts. miRNAs have pivotal functions in various biological processes including cellular differentiation, proliferation and apoptosis [11–14]. miRNAs have great potential as cancer biomarkers because of their superior stability, tissue-specificity and unique expression patterns in cancer cells [15, 16]. Expression profiling analysis has discovered a number of miRNAs deregulated in human malignancies [17, 18].

miRNAs have been identified to be crucial in tumorigenesis by playing either oncogenic or tumor suppressive roles in gastric cancer [19–23]. A number of studies have identified miRNAs that play significant roles in gastric cancer [24–30]. Our group is particularly interested in the roles of miRNAs in regulating epithelial to mesenchymal transition (EMT) in gastric cancer. Gastric epithelial cell tumor transformation is rather complex with both genetic and epigenetic involvement. Previous studies have identified several key miRNAs (miR-15a, miR-16, miR-194 and miR-200c) involved in EMT and tumor progression [31–33]. Among them, miR-15a was identified as a tumor suppressor by promoting apoptosis and inhibiting cell proliferation and downregulation of miR-15a predicts a poor survival outcome [34, 35]. Like miR-15a, down-regulation of miR-16 has also been observed to be a poor prognostic indicator [35, 36]. miR-200c has been linked with tumor progression and resistance via down-regulation of Bmi-1 in various cancers [33, 37].

One common theme is that Bmi-1 is a key target for miR-15a, miR-16, and miR-200c based on several different tumor types [31, 33, 38]. Bmi-1 is a member of the polycomb group, which functions as a transcriptional repressor and presents high expression in many tumors, in most cases, indicating a poor prognosis [38, 39]. Several lines of evidence suggest that Bmi-1 blocks cell senescence and proliferation [40, 41], and the *Bmi-1* gene is also associated with tumor invasion and metastasis [42]. Aberrant expression of Bmi-1 has been detected in several human cancers including lymphoma, acute myeloid leukemia, colorectal carcinoma, liver carcinoma, non-small cell lung cancer, breast carcinoma, prostate cancer, head and neck squamous cell carcinoma, medulloblastoma, and glioblastoma [40, 42–48]. Bmi-1 has been identified as a predictor of the response to therapy and survival in various tumors [49–51]. The miRNA mediated Bmi-1 expression and functional significance in gastric cancer remained elusive.

In this study, we systemically investigated the functional and clinical significance of miR-15a, miR-16, and miR-200c in gastric cancer. Among these, miR-15a is the most significantly down regulated miRNAs in gastric cancer. The expression of Bmi-1 is inversely correlated with the expression of miR-15a. Ectopically expression of miR-15a reduced gastric cancer cell growth and invasion. The expression of Bmi-1 is significantly correlated with gastric cancer patient survival.

## RESULTS

### Patient baseline characteristics

Table 1 summarizes the patient clinicalpathological characteristics of 352 primary gastric cancer specimens. There are 5 with stage I, 78 with stage II, 240 with stage III and 29 with stage IV gastric tumor tissues on the high density tissue microarray. Stage III samples represent 68% of all samples. Of note, there is a lack of clinical information on several patients and they have been secluded from the statistical analysis. The survival analysis revealed that the gastric cancer patients survive better with higher expression levels of Bmi-1, and correlated with smaller tumor size (< 5 cm), better pathological differentiation (I-II), less lymph node metastases (≤ 70% or earlier N stages), earlier T stages, non-metastatic disease (M0), earlier UICC stages (Table 2). The expression of Bmi-1 is significantly different in stage I-II vs. stage III-IV patients (Table 3).

**Table 1:** Correlation between patient clinicopathological characteristics and Bmi-1 expression

Characteristics	Bmi-1 expression				Total		*P*
	Lower		Higher				
	No.	%	No.	%	No.	%	
Gender							0.220
Male	173	65.3	63	72.4	236	67.0	
Female	92	34.7	24	27.6	116	33.0	
Age, years							0.157
≤ 60	120	45.5	32	21.1	152	43.3	
> 60	144	54.5	55	27.6	199	56.7	
Tumor site							0.338
Cardia	32	12.1	14	30.4	46	13.1	
Body	72	27.2	22	23.4	94	26.7	
Pylorus	126	47.5	45	26.3	171	48.6	
Unknown	35	13.2	6	14.6	41	11.6	
Tumor size ^#^							0.469
< 5 cm	107	41.6	32	37.2	139	40.5	
≥ 5 cm	150	58.4	54	62.8	204	59.5	
Tumor shape							0.481
Protrude	6	3.5	0	0	6	2.5	
Ulcerative	44	25.6	17	25.4	61	25.5	
Infiltratively ulcerative	93	54.1	39	58.2	132	55.2	
Diffuse infiltrative	17	9.9	6	9.0	23	9.6	
Superficial	9	5.2	2	3.0	11	4.6	
Colloid	3	1.7	3	4.5	6	2.5	
Pathological differentiation							0.187
I	3	1.1	2	2.3	5	1.4	
II	55	20.8	23	26.4	78	22.2	
III	181	68.3	59	67.8	240	68.2	
IV	26	9.8	3	3.4	29	8.2	
Positive rate of lymph node							0.580
≤ 70%	191	73.0	55	76.4	182	74.0	
> 70%	77	27.0	17	23.6	64	26.0	
Tumor invasion							0.092
T1	16	6.3	5	6.0	21	6.2	
T2	17	6.6	12	14.5	29	8.6	
T3	177	69.1	57	68.7	234	69.0	
T4	46	18.0	9	10.8	55	16.2	
Lymph node metastasis							0.394
N0	55	20.9	22	25.3	77	22.0	
N1	34	12.9	16	18.4	50	14.3	
N2	78	29.7	21	24.1	99	28.3	
N3	96	36.5	28	32.2	124	35.4	
Distant metastasis							0.907
M0	252	95.1	83	95.4	335	95.2	
M1	13	4.9	4	4.6	17	4.8	
UICC staging (7^th^ edition)							0.535
Stage I	12	4.7	4	4.8	16	4.7	
Stage II	74	28.9	31	37.3	105	31.0	
Stage III	157	61.3	44	53.0	201	59.3	
Stage IV	13	5.1	4	4.8	17	5.0	

# Tumor size was defined as the largest diameter of the tumor mass.

**Table 2:** Univariate analysis for survival

Characteristics	Survival time (95% CI)	Log-rank *χ*^2^	*P*
Gender		1.447	0.229
Male	31.933 (22.956 - 40.910)		
Female	26.233 (22.035 - 30.432)		
Age, years		0.651	0.420
≤ 60	29.100 (17.826 - 40.374)		
> 60	27.867 (19.884 - 35.850)		
Tumor site		0.603	0.740
Cardia	43.767 (33.309 - 54.225)		
Body	26.233 (13.621 - 38.845)		
Pylorus	31.367 (21.071 - 41.662)		
Tumor size		30.469	< 0.001
< 5 cm	50.067 (38.385 - 61.748)		
≥ 5 cm	19.333 (14.970 - 23.697)		
Tumor shape		4.031	0.133
Ulcerative	26.233 (4.483 - 47.984)		
Infiltratively ulcerative	34.867 (22.310 - 47.424)		
Diffuse infiltrative	25.033 (19.294 - 30.772)		
Pathological differentiation		12.351	< 0.001
I - II	52.767 (30.414 - 75.119)		
III - IV	25.233 (21.304 - 29.163)		
Positive rate of lymph node		54.122	< 0.001
≤ 70%	44.133 (36.234 - 52.033)		
> 70%	16.100 (11.585 - 20.615)		
Tumor invasion		50.022	< 0.001
T1	-		
T2	58.167 (35.007 - 81.326)		
T3	24.967 (21.593 - 28.341)		
T4	17.967 (13.000 - 22.934)		
Lymph node metastasis		79.468	< 0.001
N0	65.600 (52.589 - 78.611)		
N1	40.033 (30.136 - 49.931)		
N2	26.600 (17.252 - 35.948)		
N3	17.500 (14.860 - 20.140)		
Distant metastasis		9.254	0.002
M0	29.267 (21.773 - 36.760)		
M1	17.033 (14.400 - 19.667)		
UICC staging (7^th^ edition)		68.366	< 0.001
Stage I	-		
Stage II	47.867 (40.589 - 55.144)		
Stage III	20.167 (16.991 - 23.342)		
Stage IV	17.033 (14.400 - 19.667)		
Bmi-1 expression		5.091	0.024
Lower	24.533 (20.791 - 28.275)		
Higher	43.533 (30.311 - 56.756)		

**Table 3:** Different Bmi-1 expression level between gastric cancer patients with stage I-II and stage III-IV

UICC stage	No.	Bmi-1 expression level (min-max)	Z	*P*
Stage I-II	129	90 (0-290)	2.304	0.021
Stage III-IV	224	80 (0-300)		
Stage II	111	90 (0-290)	2.109	0.035
Stage III	206	80 (0-300)		

### Differential expression of miRNAs expression in gastric cancer

**Figure 1:**
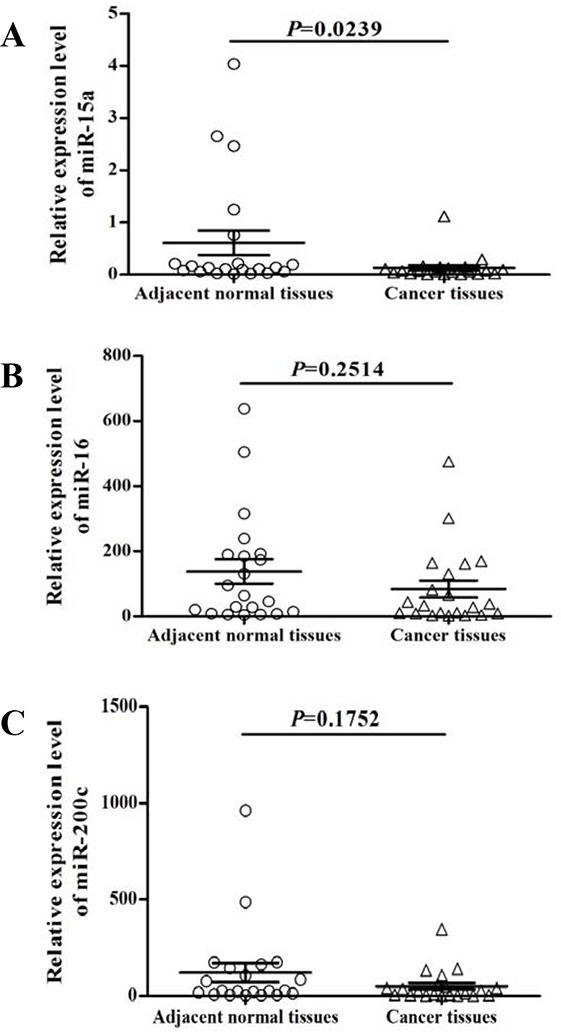
Expression analysis of miR-15a, miR-16, and miR-200c in gastric cancer The expression levels of miR-15a **A.**, miR-16 **B.** and miR-200c **C.** were quantified by qRT-PCR analysis from 21 paired normal and gastric tumor tissues. miR-15a was significantly (*P* = 0.0239) decreased in gastric cancer (A).

The expression of miRNAs (miR-15a, miR-16, and miR-200c) was quantified in 21 paired normal and gastric tumor specimens. Among these, miR-15a was significantly (*P*=0.0239) reduced in gastric cancer tissues compared to the adjacent normal controls (Figure 1A) while no significant difference in miR-16 (Figure 1B) and miR-200c (Figure 1C) were found.

### Bmi-1 is a direct target of miR-15a in gastric cancer cell lines

**Figure 2:**
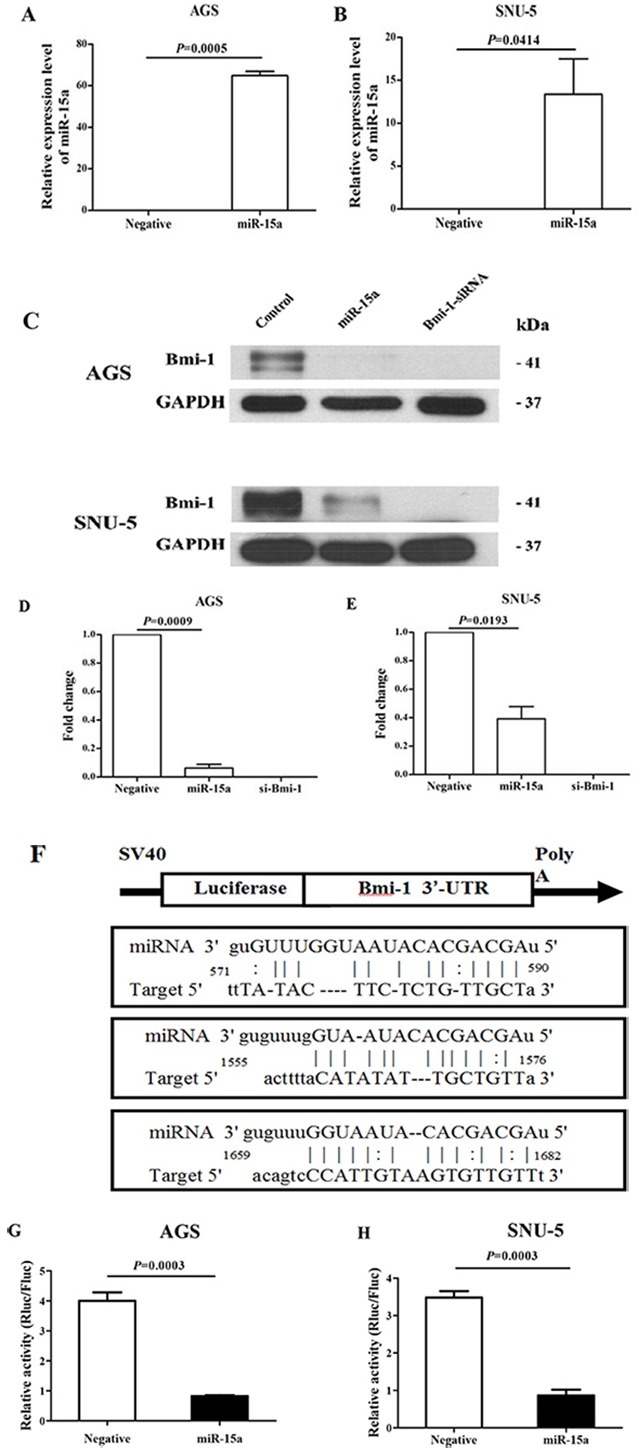
Regulation of Bmi-1 expression by miR-15a in gastric cancer cell lines qRT-PCR analysis of ectopically transfected miR-15a in AGS **A.** and SNU-5 **B.** gastric cancer cell lines. miR-15a suppresses Bmi-1 protein expression in AGS and SNU-5 cell lines by Western blotting **C.** Quantitative analysis of miR-15a in suppressing Bmi-1 expression by Western immunoblot analysis in AGS **D.** and SNU-5 **E.** cell lines. The control is a representative value of two different negative controls as both miRNA and siRNA control did not show any effect. miR-15a directly interacts with the 3′-UTR region of Bmi-1. **F.** to suppress the expression of luciferase reporter protein in AGS **G.** and SUN-5 **H.** gastric cancer cell lines.

To evaluate whether the expression of Bmi-1 is regulated by miR-15a in gastric cancer, the gastric cancer cell lines AGS and SNU-5 were transfected with 100 nM of miR-15a and negative control. The Bmi-1 protein level was quantified by Western immunoblot analysis 3 days after transfection. Our results show that we have successfully transfected miR-15a in AGS (Figure 2A) and SNU-5 (Figure 2B) based on qRT-PCR analysis. The expression of target protein Bmi-1 was significantly decreased in both AGS and SNU-5 cell lines quantified by the Western immunoblot analysis (Figure 2C) and quantified (Figure 2D–2E). There are three predicted and previous validated binding sites of miR-15a at the 3′-UTR region of Bmi-1 mRNA (Figure 2F). We have constructed a luciferase reporter system to demonstrate the direct interaction of Bmi-1 binding sites with miR-15a. Our results show that luciferase activity was significantly reduced by miR-15a in both AGS (Figure 2G) and SNU-5 (Figure 2H) gastric cancer cells.

### The expression of Bmi-1 is inversely correlated with miR-15a in gastric tumor tissues

**Figure 3:**
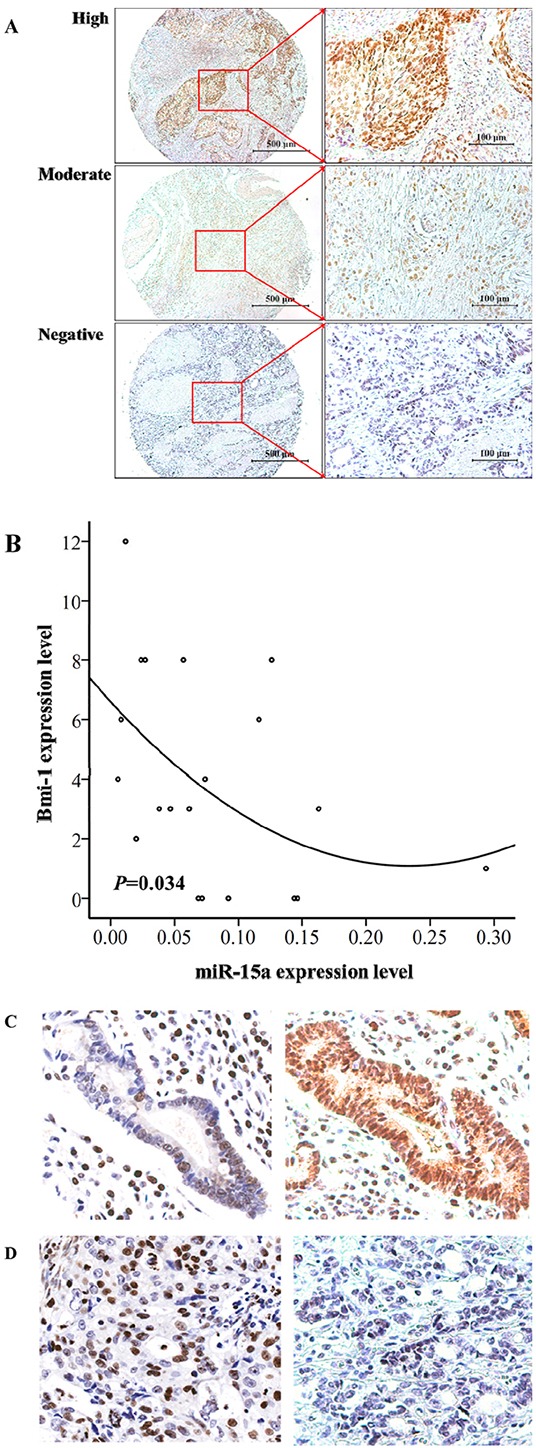
The expression level of miR-15a is inversely correlated with Bmi-1 expression in gastric tumor tissues Immunohistochemical analysis of Bmi-1 in gastric cancer tissues showing representative images of high, moderate and negative staining **A.** The expression levels of Bmi-1 is significantly (*n* = 21, *P* = 0.034, R2 = 0.22, r = −0.48) inversely correlated with miR-15a expression based on two-tailed Spearman correlation analysis. The positive rate of miR-15a is 16%. **B.** Inverse expression of miR-15a and Bmi-1 protein levels via in situ hybridization analysis of miR-15a and Bmi-1 expression quantified by IHC analysis **C–D.** in gastric tumor tissues.

The expression of Bmi-1 was quantified by IHC from 21 gastric tumor tissues. The expression of miR-15a was quantified by qRT-PCR analysis from total RNA isolated from the same set of gastric tumor tissues. The representative Bmi-1 expression ranging from negative, moderate, and high is shown in Figure 3A. The positive rate of miR-15a is about 16% of the 21 cases. The protein expression of Bmi-1 and miR-15a RNA levels was inversely correlated (*P* = 0.034, R^2^ = 0.22, r = −0.48) based on two-tailed Spearman correlation in the gastric cancer patient samples (Figure 3B). In addition, we performed *in situ* hybridization of miR-15a in gastric tumor tissues and the results are consistent with qRT-PCR analysis that elevated miR-15a staining is associated with reduced expression level of Bmi-1 by IHC (Figure 3C–3D).

### miR-15a suppressed the proliferation of gastric cancer cells via downregulation of Bmi-1

**Figure 4:**
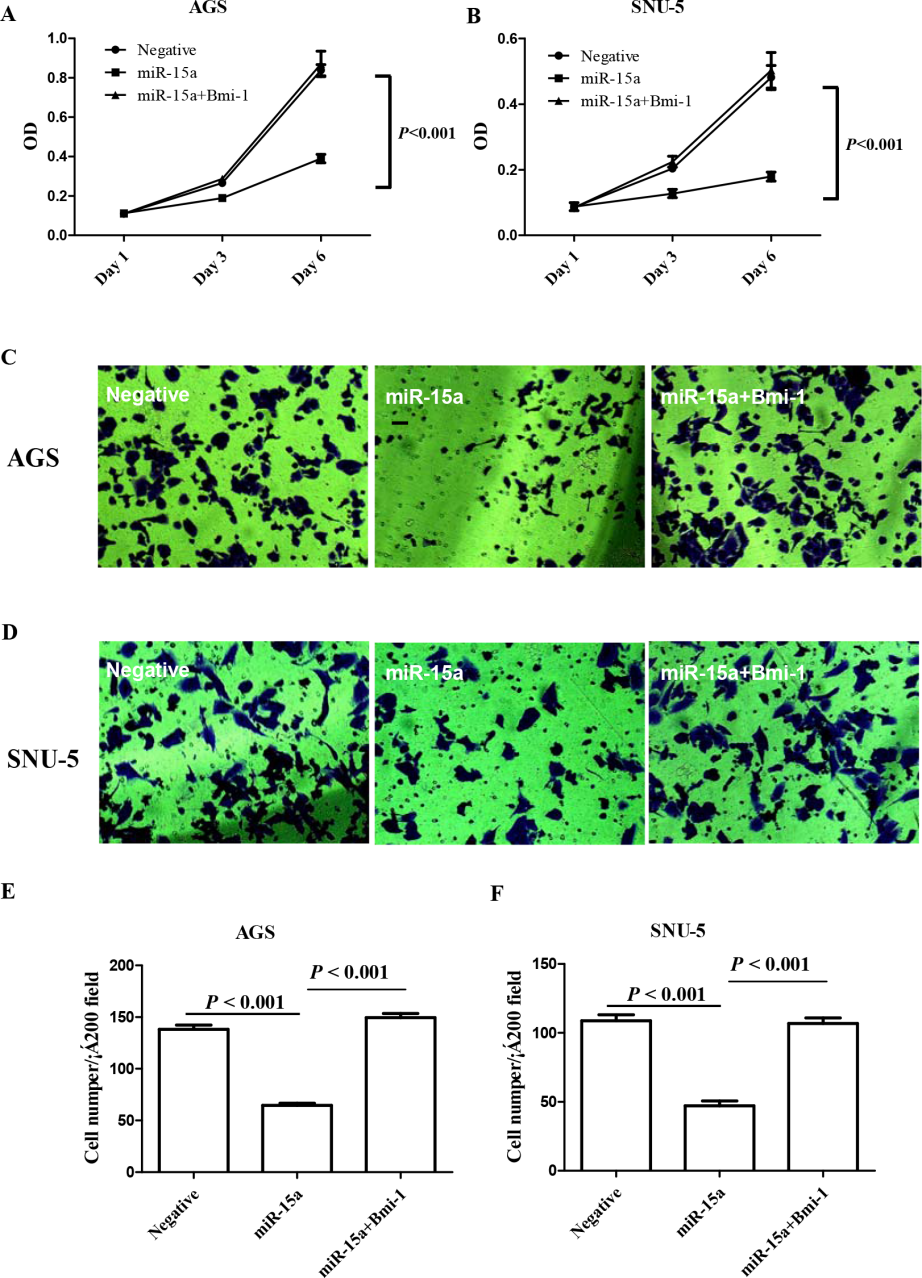
Ectopic expression of miR-15a suppressed gastric cancer cell proliferation in AGS **A.** and SNU-5 **B.** gastric cancer cell lines 3 and 6 days after transfection. Ectopic expression of miR-15a suppressed gastric cancer cell invasion in AGS **C, E.** and SNU-5 **D, F.** gastric cancer cell lines. The inhibition effect can be reversed specifically by restoration of the Bmi-1 expression.

It has been demonstrated that Bmi-1 can regulate the proliferation and clonal growth of tumor cells in a number of malignant tumors [40]. Therefore, we evaluated the role of Bmi-1 in mediating the proliferation of a gastric cancer cell lines. We found that when miR-15a was transfected into gastric cancer cell lines AGS and SNU-5, it caused a significant reduction in cell proliferation as compared to blank control (Figure 4A–4B). To ensure that such inhibition of gastric cancer cell proliferation is directly due to the reduction of Bmi-1, we were able to reverse the miR-15a suppressed proliferation by restoration of Bmi-1 expression (Figure 4A–4B).

### miR-15a suppressed gastric cancer invasion

Bmi-1regulates EMT and promotes cancer cell invasion [53], therefore we reasoned that miR-15a may have an impact on gastric cancer invasion. We used a transwell system containing an extracellular matrix (ECM) layer to study the change in invasion activity. We demonstrated that overexpression of miR-15a reduced cell number invading through the ECM by nearly 50% when compared to negative control (Figure 4C–4F).

### Association between Bmi-1 expression and patients' overall survival time and survival rates

**Figure 5:**
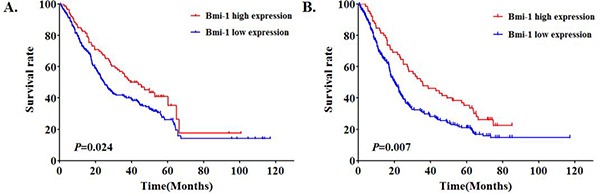
Bmi-1 expression levels are significantly associated with gastric cancer patient survival based on Kaplan-Meier survival analysis in all patients (*P* = 0.024) **A.** and in lymph node positive patients (*P* = 0.007) **B.**

We next analyzed the relationship between Bmi-1 expression and gastric cancer patients' survival based on 352 clinical samples. Based on the Kaplan–Meier survival analysis, cumulative survival curves were calculated, and differences in survival time were assessed with the log-rank test. The median survival time for gastric cancer patients demonstrating high expression of Bmi-1 was significantly (*P* < 0.024) shorter (24.5 months) in comparison to patients with low Bmi-1 expression (43.5 months) (Figure 5A). In addition, among lymph node positive gastric cancer patients, high levels of Bmi-1 patients had a significant (*P* = 0.007) better survival rate than that of patients with low levels of Bmi-1 (Figure 5B).

## DISCUSSION

In this study, we systemically investigated the functional and clinical significance of miR-15a in gastric cancer using *in vitro* and clinical samples. One of the important targets of miR-15a is Bmi-1. We quantified the expression levels of several miRNAs that were previously reported to suppress Bmi-1 protein expression in other tumor types using 25 gastric tumor specimens and the corresponding non-neoplastic gastric mucosa using qRT-PCR analysis. By comparing the expression profiles, we found a significant downregulation of miR-15a in the majority of gastric cancer tissues (Figure 1A), while there is no significant changes in the expression of miR-16 or miR-200c. We further confirmed that Bmi-1 was a direct target of miR-15a using immunohistochemical staining, qRT-PCR, luciferase reporter assay and Western blot analysis (Figure 2A–2H).

It has been reported that miR-15a inhibits cell proliferation in pancreatic ductal adenocarcinoma and ovarian cancer by downregulating Bmi-1 expression [31, 38]. Our results support the important roles of Bmi-1 played in tumor invasion and growth in gastric cancer with a unique regulatory mechanism by miR-15a.

However, despite the functional significance of Bmi-1 in regulating proliferation and progression, the clinical impact of Bmi-1 in gastric cancer is quite complex as patients with high levels of Bmi-1 had better survival than patients with low Bmi-1 expression (Figure 5A–5B). This is based on a 352 large gastric cancer patient cohort. It has been reported in breast cancer that patients with high levels of Bmi-1 had a more favorable outcome than patients with low expression of Bmi-1 [54]. This is different from the findings in other gastrointestinal tumor types such as colorectal cancer and pancreatic cancer, where patients with low Bmi-1 expression had a better survival outcome [38, 55]. This discrepancy might be explained by the complex function of polycomb protein Bmi-1. Bmi-1 functions as a double edged sword. It has been reported that leukemic stem cells lacking Bmi-1 do not proliferate [56]. As a transcriptional repressor, Bmi-1 also regulates cell proliferation and senescence through the ink4a locus [40]. As a result, gastric tumor cells that have low levels of Bmi-1 will be slowly proliferating and insensitive to chemotherapy which target rapid proliferative cells. In contrast, gastric cancer cells with high levels of Bmi-1 proliferate rapidly, making them sensitive to chemotherapy. This is consistent with our observation that knock-down Bmi-1 expression by miR-15a reduces gastric cancer cell proliferation (Figure 4A–4B). Our results suggest that there is a good possibility that a relative large number of patients retained a significantly high enough level of miR-15a to suppress the expression of Bmi-1 in gastric cancer. The favorable outcome of gastric cancer patients with high levels of Bmi-1 may be due to the fact that these gastric tumor cells are highly sensitive to chemotherapy treatment while patients with low Bmi-1 level are less sensitive. Another possibility is that high levels of Bmi-1 are crucial in blocking MET process of advance stage gastric tumor *in vivo*. Such phenotypes warrant further investigation.

In summary, we discovered that the expression of Bmi-1 is regulated by miR-15a in gastric cancer. The prognostic significance of Bmi-1 in gastric cancer supports future multi-center large cohort studies.

## MATERIALS AND METHODS

### Patients and samples

A total of 21 formalin-fixed paraffin-embedded (FFPE) gastric cancer tissues and the paired normal tissues were obtained from the Third Affiliated Hospital of Soochow University, China. Written patient consent was obtained and the study was approved by the Ethics Committee of the Third Affiliated Hospital of Soochow University. Human gastric cancer high density tissue microarray from Shanghai Outdo Biotech Co. LTD. contains 352 primary tumor specimens with clinical follow up information included for the analysis of Bmi-1 expression and survival analysis. The patient baseline characteristics are listed in Table 1. Of note, some of the patient's clinical information is not available and they were secluded from the statistical analysis.

### Cell lines and culture conditions

The gastric cancer cell lines AGS and SNU-5 were obtained from American Type Culture Collection (ATCC), and were maintained in F-12K supplemented with 10% fetal bovine serum and IMDM supplemented with 20% fetal bovine serum medium respectively (Life Technologies Inc.).

### miRNA transfection

Gastric cancer cell lines AGS and SNU-5 were cultured in appropriate medium for 24 h before the transfection experiment. Using Lipofectamine™ 2000 (Life Technologies Inc.), the cell line was transfected with 100 nM of miR-15a or negative control. The Bmi-1 protein expression was quantified 72 h post-transfection by Western blotting.

### RNA and protein isolation

Total RNA from the transfected gastric cancer cell lines as well as frozen gastric cancer samples was extracted using TRIzol reagent, and total protein was isolated from the transfected cell lines using RIPA buffer (Sigma-Aldrich). The RNA and protein extractions were stored at −80°C.

### Western immunoblot analysis for Bmi-1 expression

Seventy-two hours after transfection, cells were lysed with RIPA buffer (Sigma-Aldrich), and Western immunoblot analysis was performed using standard procedures. The primary antibodies used for the analysis were mouse anti-human Bmi-1 antibody (1:2000; Cell Signaling), mouse anti-human GAPDH antibody (1:2000, Santa Cruz Biotechnology, Santa Cruz, CA, USA). Horseradish peroxidase-conjugated (HRP) antibodies against mouse (1:5000; Bio-Rad, Hercules, CA, USA) or against rabbit (1:5000; Cell Signaling Technology) were used as the secondary antibodies. HRP activity was detected with SuperSignal West Pico Chemiluminescent Substrate (Thermo Fisher Scientific) and visualized using autoradiography film. Quantification was performed using ImageJ software.

### Expression analysis of miRNAs by real-time qPCR

FFPE gastric tumor tissues were deparaffinized, hydrated, and digested with proteinase K (Sigma–Aldrich). Subsequently, total RNA was isolated using the TRIzol reagent (Life Technologies Inc.). Total RNA was also isolated from the clinical specimens with the TRIzol-based approach. The candidate miRNAs (miR-15a, miR-16 and miR-200c) shown previously to regulate Bmi-1 were quantified using real-time qRT-PCR TaqMan microRNA analysis in both normal and gastric cancer samples.

The miRNA-specific primers and the internal control RNU44 gene primers were purchased from Life Technologies. cDNA synthesis was performed by the High Capacity cDNA Synthesis Kit (Life Technologies Inc.) with miRNA-specific primers. Real-time qRT-PCR was carried out on an Applied Biosystems 7500 Real-Time PCR machine with miRNA-specific primers by TaqMan Gene Expression Assay (Life Technologies Inc.). Expression level of miR-15a was calculated by the ΔΔCt method based on the internal control RNU44, normalized to the control group and plotted as relative quantification.

### Expression analysis of miR-15a by in situ hybridization

The expression analysis of miR-15a in FFPE gastric tumor tissues were quantified by *in situ* hybridization performed by BioGenex Inc. (Fremont, CA). In brief, 5′-fluorescein-labeled LNA-modified DNA oligonucleotides against the full length of the miR-15a were used for ISH analysis [52]. Probes with mismatch mutations were used as control. An antibody sandwich detection of miRNA probe using anti-fluorescein primary antibody followed by a tyramide signal amplification reaction, in which HRP conjugated to secondary antibody activates the tyramine moiety of a fluorochrome-conjugated substrate resulting in a covalent attachment of this fluorescent reagent to proteins in the vicinity of the miRNA probe. The signals were detected by the BioGenex's imaging detection system.

### Cell proliferation assay

Twenty-four hours after transfection, cells were seeded in 96-well plates at a density of 2000 cells per well. The cell proliferation assay was performed on days 1, 3 and 6 by incubating 10 ml WST-1 (Roche Applied Science) in the culture medium for 1 h and reading the absorbance at 450 and 630 nm. The cell proliferation rate was calculated by subtracting the absorbance at 450 nm from the absorbance at 630 nm.

Experiments for the cell proliferation assay were performed at least three times.

### Immunohistochemistry (IHC)

IHC analysis of Bmi-1 was performed using a standard streptavidin–biotin-peroxidase complex method. For antigen retrieval, paraffin tissue slides were microwave-treated for 2 h and boiled in a 0.01 M citrate buffer (PH = 6.0) for 18 min. The slides were the treated with 0.5% Triton for 20 min to break the cell membranes. The slides were incubated with mouse mAb against human Bmi-1 (Millipore, 1:200 dilution) overnight at 4°C and then incubated with goat anti-mouse

IgG for 2 h. Normal pancreatic tissue slides were acquired by superseding the primary antibody with normal rabbit or mouse IgG.

### Statistical analysis

All data were analyzed with SPSS statistical software (SPSS Standard version 13.0, SPSS Inc.). For univariate survival analysis, we analyzed all gastric cancer patients by Kaplan–Meier analysis. The log-rank test was used to compare different

survival curves. The association between Bmi-1 protein expression and the clinicopathological features of the gastric cancer patients was assessed with the Chi-square test. *P* < 0.05 was considered statistically significant. All statistical analyses were performed with Graphpad Prism (version 6.01) software (La Jolla, CA, USA). The statistical significance between two groups was determined by Wilcoxon matched-pairs signed-rank test for clinical samples and by Student's unpaired t-test for all other experiments. The statistical significance among several groups was analyzed by Kruskal–Wallis oneway analysis of variance test with Dunn's multiple comparisons test. Data were expressed as mean ± S.E.M.
